# Mek inhibition results in marked antitumor activity against metastatic melanoma patient-derived melanospheres and in melanosphere-generated xenografts

**DOI:** 10.1186/1756-9966-32-91

**Published:** 2013-11-16

**Authors:** Giovanni Sette, Katia Fecchi, Valentina Salvati, Fiorenza Lotti, Emanuela Pilozzi, Enrico Duranti, Mauro Biffoni, Alfredo Pagliuca, Daniela Martinetti, Lorenzo Memeo, Michele Milella, Ruggero De Maria, Adriana Eramo

**Affiliations:** 1 Department of Hematology, Oncology and Molecular Medicine, Istituto Superiore di Sanità, viale Regina Elena 299, Rome 00161, Italy; 2 Regina Elena National Cancer Institute, Rome, Italy; 3 Department of Clinical and Molecular Medicine, Sant’Andrea Hospital, University ‘La Sapienza’, Rome, Italy; 4 Mediterranean Institute of Oncology, Catania, Italy

**Keywords:** Metastatic Melanoma, Mek inhibition, Melanospheres, Target therapy

## Abstract

One of the key oncogenic pathways involved in melanoma aggressiveness, development and progression is the RAS/BRAF/MEK pathway, whose alterations are found in most patients. These molecular anomalies are promising targets for more effective anti-cancer therapies. Some Mek inhibitors showed promising antitumor activity, although schedules and doses associated with low systemic toxicity need to be defined. In addition, it is now accepted that cancers can arise from and be maintained by the cancer stem cells (CSC) or tumor-initiating cells (TIC), commonly expanded *in vitro* as tumorspheres from several solid tumors, including melanoma (melanospheres). Here, we investigated the potential targeting of MEK pathway by exploiting highly reliable *in vitro* and *in vivo* pre-clinical models of melanomas based on melanospheres, as melanoma initiating cells (MIC) surrogates. MEK inhibition, through PD0325901, provided a successful strategy to affect survival of mutated-BRAF melanospheres and growth of wild type-BRAF melanospheres. A marked citotoxicity was observed in differentated melanoma cells regardless BRAF mutational status. PD0325901 treatment, dramatically inhibited growth of melanosphere-generated xenografts and determined impaired tumor vascularization of both mutated- and wild type-BRAF tumors, in the absence of mice toxicity. These results suggest that MEK inhibition might represent a valid treatment option for patients with both mutated- or wild type-BRAF melanomas, affecting tumor growth through multiple targets.

## Introduction

Melanoma is one of the most aggressive cancers, with increasing incidence worldwide [1,2]. Currently available cytotoxic treatment options produce low rates of patient response and have modest survival impact. Therefore, there is an urgent need for development of more effective therapies that may rely on molecularly targeted individualized treatments. One of the key oncogenic pathways most frequently altered in melanoma is the RAS/BRAF/MEK pathway, thus providing potential promising therapeutic targets [3-7]. Specific inhibitors have been developed, partially investigated *in vitro* and some of them entered clinical trials [8-10]. Recent melanoma patient improvement has been observed using targeted therapy or immunotherapy. Indeed, the BRAF inhibitor, vemurafenib, and anti cytotoxic T-lymphocyte antigen 4 (CTLA-4) antibody, ipilimumab, demonstrated a survival benefit [11,12]. Despite the success of these treatments, most patients eventually progress. In addition, BRAF regulatory loops may circumvent its inhibition, thus Mek, being downstream of BRAF in this key molecular pathway, may represent a highly relevant clinical target [10,13,14]. Currently, thirteen MEK inhibitors, including trametinib, pimasertib, refametinib, PD-0325901, TAK733, MEK162 (ARRY 438162), RO5126766, WX-554, RO4987655 (CH4987655), GDC-0973 (XL518), and AZD8330 have been tested clinically but only trametinib (GSK1120212), a selective inhibitor of MEK 1 and 2, has emerged as the first MEK inhibitor to show favorable clinical efficacy in a phase III trial in BRAF mutated melanoma. It is being evaluated by FDA for the treatment of metastatic melanoma with BRAF V600 mutation. Finally, several clinical trials are currently ongoing using MEK inhibitors in combination with chemotherapeutic drugs (including dacarbazine or paclitaxel). However, schedules and doses of Mek inhibitors compatible with satisfactory antitumor efficacy associated with low systemic toxicity need to be further defined [15-19]. On the other hand, it would be relevant to determine whether the pathway signature of the bulk tumor characterizes also the melanoma initiating cell (MIC) compartment in order to favor potentially more curative MIC-effective molecularly targeted approaches [20-22]. In fact, increasing experimental evidence supports the assertion that many tumors including melanomas, contain Cancer Stem Cells (CSC) or Tumor-Initiating Cells (TIC) and that they affect tumor biology, thus acquiring dramatic clinical relevance [4,20,23]. This course has triggered emerging interest and important studies have been performed in the attempt to understand the nature of MIC. Several putative MIC markers have been identified including CD20, CD133, ABCB5, CD271, JARIDB1, ALDH, however most of these markers have not yet been validated in independent studies [24-35]. Intense debate in this field is on-going and, to date, several controversies surrounding this field remain unsolved, including those concerning the frequency of MIC. [29,30,35-38]. Extending beyond the general view that CSC are static entities, recent evidence support a model of dynamic stemness in which tumor maintenance, in some solid tumors, may be a dynamic process mediated by a temporarily distinct sub-population of cells that may transiently acquire stemness properties and continually arise and disappear (“moving target”) depending on the tumor context, with consequent therapeutic implications [30,32,37-39]. However, even though their frequency, phenotype and nature still remain controversial issues, the existence of a sub-population of cells with increased tumor-initiating potential in melanomas is not questioned [40].

We investigated the activation and potential targeting of the MEK pathway, exploiting highly reliable *in vitro* and *in vivo* pre-clinical models of melanomas based on melanospheres. We isolated the highly tumorigenic cell sub-population from patient metastatic melanomas based on its functional ability to grow indefinitely as melanospheres. We previously proved that this approach efficiently enriches tumorigenic cells *in vitro*[41-44]. Given that this strategy did not rely on any prospective cell separation based on putative CSC-markers, it allowed us to overcome the possible bias of selecting cell populations based on the presence of transiently expressed antigens. The availability of exponentially growing melanospheres allowed us to obtain their deep *in vitro* validation and develop preclinical therapeutic approaches to target both the more tumorigenic and bulk tumor cell populations *in vitro* and *in vivo.*

## Materials and methods

### Ethics statement

Tumor samples were obtained in accordance with consent procedures approved by the Internal Review Board of Sant’ Andrea Hospital, University ‘La Sapienza’ , Rome, Italy. All patients signed an informed consent form.

According to the Legislative Decree 116/92 which has implemented in Italy the European Directive 86/609/EEC on laboratory animal protection, the research protocol “Analysis of effectiveness and tolerability of anti-tumor therapeutic agents in mice carrying cancer stem cell-derived tumors” (Principal Investigator Dr. Adriana Eramo) has been approved by the Service for Biotechnology and Animal Welfare of the Istituto Superiore di Sanità and authorized by the Italian Ministry of Health (Decree n° 217/2010-B). The animals used in the above mentioned research protocol have been housed and treated according to Legislative Decree 116/92 guidelines, and animal welfare was routinely checked by veterinarians from the Service for Biotechnology and Animal Welfare.

### Isolation and culture of melanospheres and obtainment of differentiated progeny

Tumor samples were obtained in accordance with consent procedures approved by the Internal Review Board of Department of Laboratory Medicine and Pathology, S. Andrea Hospital, University La Sapienza, Rome. Surgical specimens were dissociated and recovered cells cultured in serum-free medium as previously described [41,42]. Briefly, surgicalspecimens were washed several times and left over night in DMEM:F-12 medium supplemented with high doses of Penicillin/Streptomycin and Amphotericin B in order to avoid contamination. Tissue dissociation was carried out by enzymatic digestion (1.5 mg/ml collagenase II, Gibco-Invitrogen, Carlsbad, CA and 20 μg DNAse I, Roche, Mannheim, Germany) for 2 hours at 37°C. Recovered cells were cultured in serum-free medium containing 50 μg/ml insulin, 100 μg/ml apo-transferrin, 10 μg/ml putrescine, 0.03 μM sodium selenite, 2 μM progesterone, 0.6% glucose, 5 mM hepes, 0.1% sodium bicarbonate, 0.4% BSA, glutamine and antibiotics, dissolved in DMEM-F12 medium (Gibco-Invitrogen, Carlsbad, CA) and supplemented with 20 ng/ml EGF and 10 ng/ml bFGF. Flasks non-treated for tissue culture were used in order to reduce cell adherence and support growth as undifferentiated tumor-spheres. Medium was replaced or supplemented with fresh growth factors twice a week until cells started to grow forming floating aggregates. Cultures were expanded by mechanical partial dissociation of spheres, followed by re-plating of cells and residual small aggregates in complete fresh medium. *In vitro* differentiation was obtained by melanosphere cell culture in Melanocyte Growth Medium (MGM4, Lonza, East Rutherford, NJ, USA). Melanocytes (Lonza) were cultured in the same conditions. Alternatively, differentiated cells were obtained from standard (DMEM + 10% FBS) culture of tumor cells obtained from mouse xenografts.

### Immunohistochemistry on tumor sections

Immunohistochemistry was performed on formalin-fixed paraffin-embedded or frozen tissue. Five μm paraffin sections were dewaxed in xylene and rehydrated with distilled water. Sections were treated with the heat-induced epitope retrieval technique using a citrate buffer (pH6). After peroxidase inhibition with 3% H_2_O_2_ for 20 minutes, the slides were incubated with the following antibodies: anti Phospho-p44/42 MAPK (Erk1/2) (Cell Signaling Beverly, Ma, USA), anti MART-1, S100 and KI-67 (DAKO, Glostrup, Denmark), anti CD34 (Rat monoclonal, clone 14.7, Novus Biologicals), anti-VEGF (rabbit polyclonal, A20, Santa Cruz). The reaction was performed using Elite Vector Stain ABC systems (Vector Laboratories) and DAB chromogen substrate (DakoCytomation), followed by counterstaining with haematoxylin.

### Chemotherapy and PD0325901 treatment

Three thousand cells obtained from melanosphere dissociation were plated in 96-well flat-bottom plates. Chemotherapeutic agents were added at the following final concentrations: paclitaxel 30 ng/ml, cisplatin 5 μg/ml, dacarbazine 5 μg/ml and temozolomide 100 μM and Mek inhibitor PD0325901 (Pfizer) 200nM. Cell viability was evaluated after a 2 day treatment with chemotherapic agents or a 3 day treatment with PD0325901 by both luminescent cell viability assays (CellTiter-Glo, Promega, Madison, WI, USA) and cell count by trypan blue exclusion. Data represented are means of three independent experiments performed by the two experimental procedures.

### Western blot

Proteins were resolved on 4-12% polyacrylamide gel electrophoresis NuPAGE Bis-Tris (Invitrogen, Carlsbad, CA) and transferred to nitrocellulose membranes. Rabbit polyclonal anti-Phospho-S6 (Ser240/244) were purchased from Cell Signaling (Beverly, Ma,USA), mouse monoclonal anti-Phospho-ERK (clone E-4) and anti-p16 (clone JC8), rabbit polyclonal anti-cyclin D1 (M20), anti-VEGF (A20) and anti-Erk (K23) were purchased from Santa Cruz (Santa Cruz, Ca, USA). β-Tubulin was purchased from Sigma-Aldrich (St. Louis, Mo, USA). Anti-mouse or anti-rabbit horseradish peroxidise-conjugated secondary antibodies were purchased from Amersham Pharmacia Biotech (Buckinghamshire, UK).

### Inhibitors screening

Eighty inhibitors targeting different survival pathways (Enzo Life Sciences, New York, NY, http://www.enzolifesciences.com) were tested on 3 different melanospheres samples, at the final concentration of 5 μM. Cell viability was evaluated after 2 days of treatment by luminescent cell viability assay (CellTiter-Glo, Promega, Madison, WI, USA).

### Cell cycle analysis and apoptosis assay

For cell cycle assay 1 × 10^5^ cells were washed with PBS and suspended in Nicoletti buffer (0.1% sodium citrate, pH 7.4/0.1% Triton X) containing 100 μg/ml propidium iodide and 200 μg/ml RNaseA. After 2 hrs of incubation at 4°C, samples were analyzed with FACS Canto (Becton Dickinson, Franklin Lake, NJ, USA).

Apoptosis was measured using the Apoptosis Detection Kit I (BD Bioscience). One million cells/ml were stained with 5 μl of Annexin V-FITC (BD PharMingen) and 10 μg/ml 7AAD (Sigma-Aldrich, St. Louis, Mo, USA) in a total volume of 100 μl and analyzed by FACS Canto.

### Xenograft generation and mice treatment

The research protocol “Analysis of effectiveness and tolerability of anti-tumor therapeutic agents in mice carrying cancer stem cell-derived tumors” (P.I. Dr. Adriana Eramo) has been approved by the Service for Biotechnology and Animal Welfare of the Istituto Superiore di Sanità and authorized by the Italian Ministry of Health (Decree n° 217/2010-B).

Melanospheres were injected in complete medium:Matrigel (BD Pharmingen) in the flank of four to six week-old female NOD-SCID or nude mice (Charles River). Once tumor diameters reached a maximum of 10 mm, mice were sacrificed, tumor tissues collected, fixed in buffered formalin and analyzed by immunohistochemistry. For drug experiments, when tumors reached a mean of 0.5 cm in diameter, mice were randomized into 3 groups. One group was left untreated and the others were treated for 3 weeks with 12.5 mg/Kg or 25 mg/Kg of PD0325901 (freshly dissolved in 0.5% hydrossimethylcellulose/0.2% tween80) administered orally by gavage on day 1 and day 4 of each week. Tumors were measured twice a week for the 3 weeks using a caliper, and mice were monitored for signs of drug-induced toxicity and weighed with similar schedules. At the end of treatment tumors werefixed in formalin and embedded in paraffin for IHC or frozen at -80°C for protein lysates. Protein lysates were obtained homogenizing three times at high speed (Polytron model 200, Pro Scientific Inc.) at 4°C for 20 minutes in a homogenizing solution containing 10 mM Tris pH 8.0, 150 mM NaCl, 1 mM EDTA, 1 mM orthovanadate, 1% Triton X-100, and 60 mM N-octyl-b-D-glucopyranoside, in the presence of protease inhibitors. After 10 min of centrifugation (13,000 rpm, 4°C), protein concentration was determined by the Bradford assay (Biorad).

### Statistical analysis

Results are expressed as means ± S.D: Statistical calculations were performed with Microsoft Excel analysis tools. Comparisons between means were performed by Student’s t test, and the P < 0.05 was regarded as significant.

## Results

### Melanospheres isolated from metastatic melanoma tumors possess stem cell properties, are highly tumorigenic in vivo and recapitulate the patient tumor

**Figure 1:**
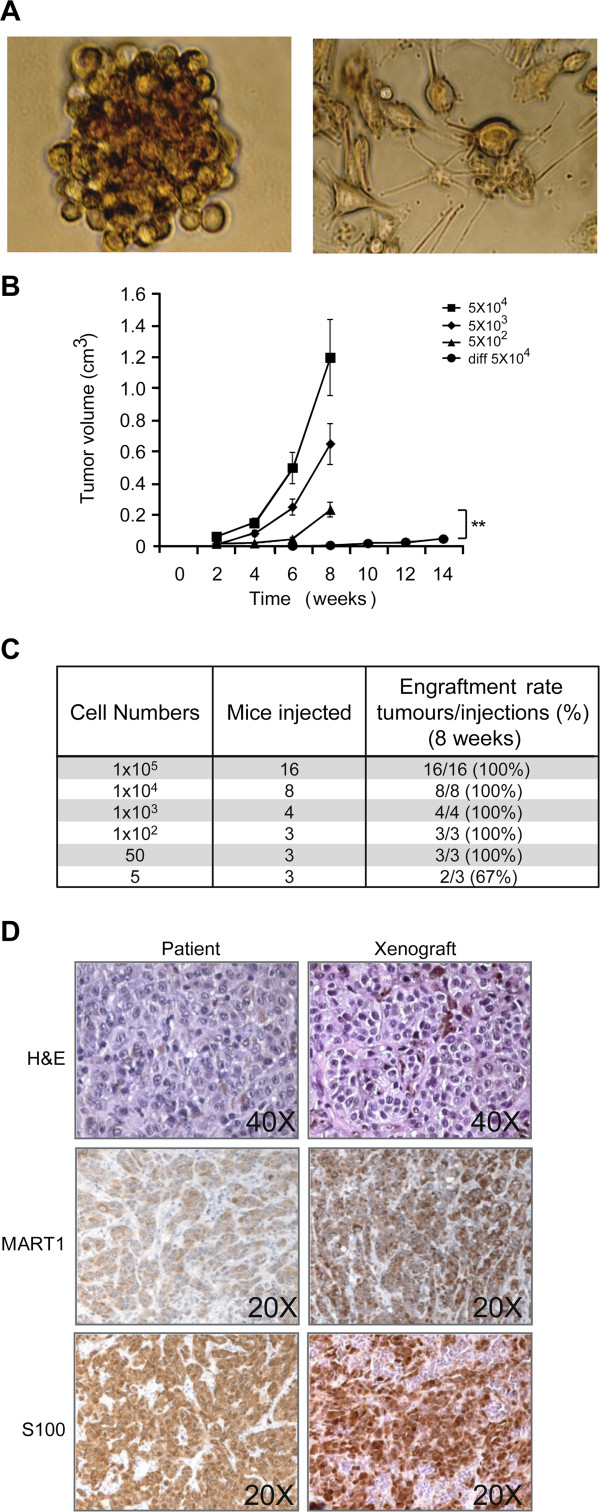
**Melanosphere isolation and validation. A)** Image of melanospheres (left) and their differentiated progeny (right). **B)** Tumor volumes of xenografts generated by spheres or differentiated (diff) melanoma cells injected subcutaneously in Nude mice at the indicated cell doses. Mean ± SD of 3 independent experiments is shown. ** p < 0,01. **C)** Table of melanospheres tumorigenicity in dose response experiments. Cell numbers, number of mice injected and percentage of tumor engraftment is indicated for each condition. Tumors were monitored for 8 weeks post-injection. **D)** Hematoxylin and eosin (H&E) or immunohistochemistry for the indicated antigens performed on patient tumor or xenograft generated by melanospheres. The original magnification of each image is indicated.

Ten patient-derived metastatic melanoma specimens were enzymatically dissociated and tumor cells were cultured in selective conditions for CSC (tumor spheres), as previously described [41-44]. Following prolonged culture, we obtained exponentially growing “melanospheres” with efficiency of 80% (Figure 1A left). The same cells cultured in conditions specific for the growth of melanocytes generated monolayers of tumor cells whose morphology resembled differentiated cells, suggesting the capacity of melanospheres to differentiate *in vitro* (Figure 1A right).

We next investigated the expression of antigens that have been previously associated with MIC. Melanospheres did not express CD133, CD20, CD24, ABCB5 or CD271 (Additional file 1: Figure S1A-B), while p-glycoprotein was detectable at low levels. They expressed stem cell-related markers as c-Kit, Cripto, CD146, CD44 and CD166 (Additional file 1: Figure S1A) in agreement with previous reports on cell line-derived melanospheres [38]. Finally, embryonic stem cell markers Nanog and Oct-4 were detected at the RNA level in all samples analyzed (Additional file 1: Figure S1C). The CD44 isoform V6 was specifically restricted to melanospheres, being not expressed in differentiated cells, nor in tumor cells freshly isolated from melanosphere-derived xenografts nor in melanocytes (Additional file 1: Figure S1D).

Melanospheres could be expanded *in vitro* for several months and their proliferation rate was not lost with time (Additional file 2: Figure S2A). They were composed by a large (mean 42% ± 8 in all examined samples) fraction of self-renewing sphere-reforming cells (Additional file 2: Figure S2B upper left). Finally, secondary and tertiary spheres were formed with a similar frequency and tertiary spheres were able to proliferate indefinitely, indicating that the fraction of self-renewing cells did not decrease with passages (Additional file 2: Figure S2B upper right panel). The clonogenic activity was higher in melanospheres than in their differentiated counterpart (Additional file 2: Figure S2B lower panels). Under appropriate conditions, melanospheres generated a progeny of cells with morphology and phenotype of melanocytic, adipogenic or osteogenic cells, demonstrating multiple differentiation ability and recapitulating the plasticity of neural crest cells (Additional file 2:Figure S2C).

Melanospheres were highly tumorigenic when injected subcutaneously in NOD Scid or Nude mice and all samples displayed tumor take of 100% down to 25000 cells. For one sample we performed a limiting dilution experiment and even as low as 5 cells readily generated a tumor within 8 weeks (Figure 1B and C). In contrast, melanosphere-derived differentiated cells displayed a decreased and delayed tumor growth *in vivo*, and as many as 5x10^4^ differentiated cells generated a slowly growing tumor with a 10-week delay post-injection (Figure 1B). Immunohistochemical analysis of melanosphere-derived xenografts, performed for all samples, revealed a high similarity between the xenograft and the original patient tumor in terms of morphology and expression of the melanoma-associated diagnostic antigens MART1 and S100 (Figure 1D is a representative image). Following xenograft dissociation and re-injection we easily obtained secondary and tertiary tumors, suggesting that tumorigenic potential was not lost with passages in mice, in fact these results proved the ability of tumorigenic cells to self-renew *in vivo* (results not shown). Based on these *in vitro* and *in vivo* results, we considered melanospheres as surrogate of melanoma-initiating cells (MIC) exploitable for pre-clinical experimentation.

### Melanospheres are resistant to chemotherapeutic drugs and to most pathway inhibitors

**Figure 2:**
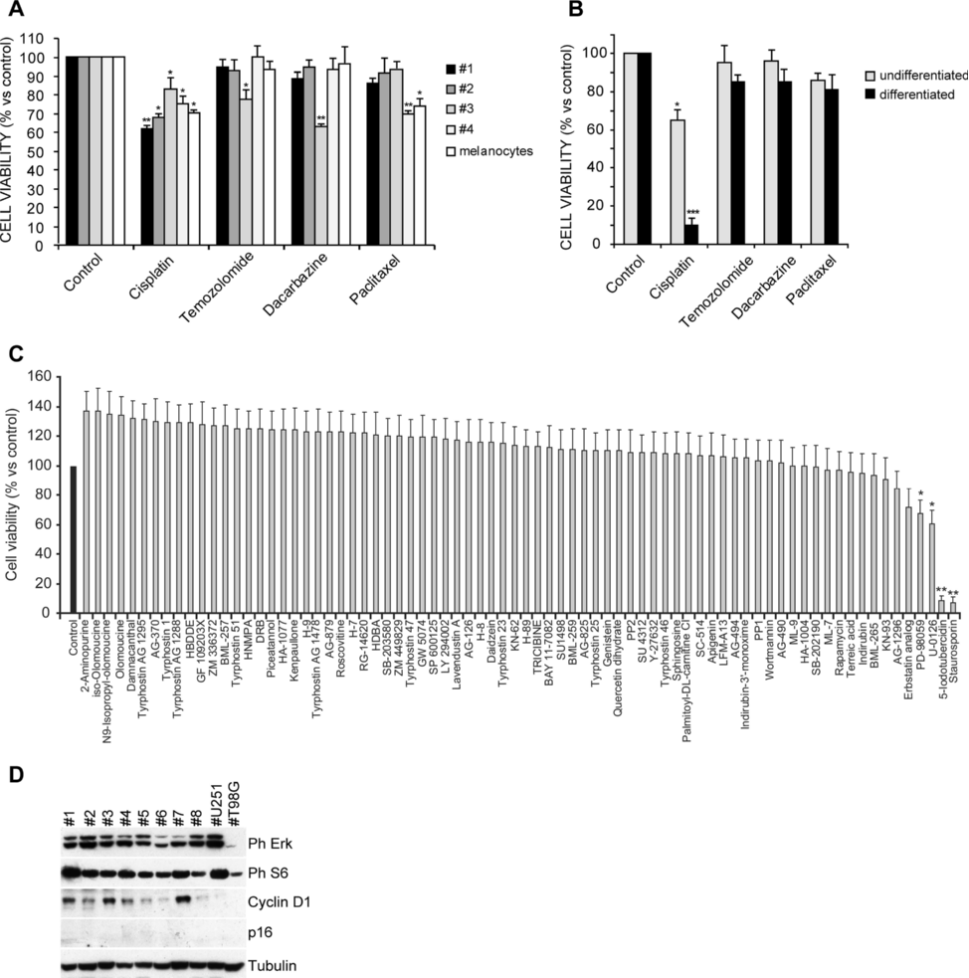
**Drug resistance of melanosphere and pathway activation. A)** Cell viability of undifferentiated melanospheres of the indicated samples and melanocytes treated with the indicated drugs. Mean ± SD of 3 independent experiments is shown. ** p < 0,01. **B)** Cell viability of melanospheres (undifferentiated) and their progeny (differentiated) exposed to the indicated chemotherapeutic agents. A representative sample is shown. Mean ± SD of 3 independent experiments is shown. *** p < 0,001. **C)** Cell viability of melanospheres exposed to the indicated kinase inhibitors. Mean ± SD of 3 independent experiments is shown. ** p < 0,01; * p < 0,05 **D)** Immunoblot analysis of the indicated proteins or phosphoproteins in melanospheres. U251 and T98G glioblastoma cell lines were used as p-ERK positive and negative control, respectively.

We investigated the response of melaospheres to chemotherapeutic agents currently used in the treatment of melanoma patients. Melanospheres were exposed to cisplatin, temozolomide, dacarbazine and paclitaxel for 48 hours and cell viability was assessed by MTT assay. Overall a weak cytotoxic effect (<40% in all samples and with all drugs) was observed with no therapeutic window as compared to normal melanocytes (Figure 2A). Conversely, differentiated cells were extremely sensitive to cisplatin, in 3 out of 3 samples assessed (Figure 2B is a representative sample).

We next investigated the cytotoxic potential of a panel of 80 signaling pathway inhibitors on melanospheres derived from patient #1 and #2 and #3 encompassing inhibitors of RAS/RAF/MEK and PI3K/AKT pathways as well as tyrosine kinase receptors. Only inhibitors of the RAS/RAF/MEK pathway (including the MEK inhibitors PD098059 and U0126 and the Erk2 inhibitor 5-iodotubercidin) showed promising antitumor activity in terms of reduced cell viability, as measured by MTT assay. The other drugs, except for the broadly toxic compound staurosporin used as positive control, were nearly unable to reduce cell viability/proliferation, although all compounds were used at doses higher than the described IC_50_ in order to enhance their activity. A similar drug response was observed for the different samples (Figure 2C shows a representative one). In line with the melanosphere sensitivity to compounds targeting the MAPK pathways, we observed the activation of this signaling pathway with high levels of phosphorylation of Erk and downstream S6 (Figure 2D). We also found high levels of Cyclin-D and undetectable p16 (Figure 2D). These results are in agreement with the frequent alteration of the RAS/RAF/MEK pathway and cell cycle deregulation found in melanomas. Next, we analyzed DNA sequences of genes whose alterations may contribute to the abnormal pathway activation. As reported in the Additional file 3: Table S1, NRAS was never mutated in the analyzed samples. Instead, despite the ubiquitous Erk phosphorylation found in melanospheres, the BRAF-V600E mutation was detected in samples 1, 2 and 4, BRAF-V600K mutation was found in samples 5 and 8, while samples 3, 6 and 7 displayed wild type BRAF. All samples displayed wild type PTEN. Finally, sequence analysis of the exon 4 and 5 of GNAQ gene, whose mutations have been associated with wild type BRAF and NRAS melanomas, revealed wild type status in all samples (Additional file 3: Table S1 and Additional file 4) [45].

### Treatment with MEK inhibitor PD0325901 results in strong antitumor activity against melanospheres

**Figure 3:**
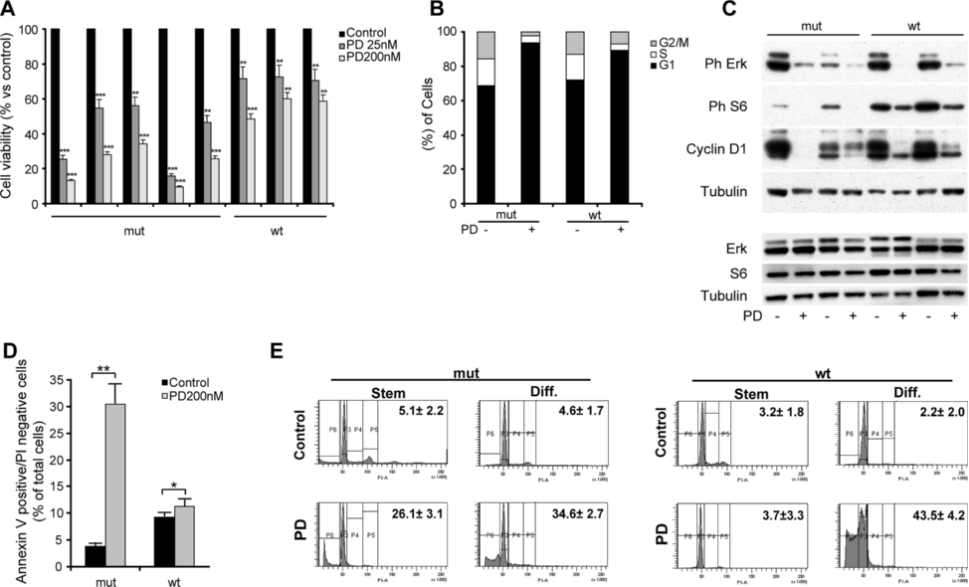
**Antitumor activity of PD0325901 on melanospheres and their progeny. A)** Cell viability (Cell Titer Glo assay, Promega) of melanospheres with mutated- or wild type-BRAF treated with the indicated drug doses. Mean ± SD of 3 independent experiments is shown. *** p < 0,001. Cell cycle distribution **(B)** and immunoblot analysis of pathway activation **(C)** of melanospheres after a 2 day drug exposure. **D)** Percentage of AnnexinV positive cells in control or PD0325901-treated representative melanospheres samples with mutated- or wild type-BRAF. Mean ± SD of 3 independent experiments is shown. ** p < 0,01. **E)** Propidium Iodide staining and flow cytometric analysis of representative samples of melanospheres (stem) or differentiated (diff) melanoma cells with mutated- or wild type-BRAF untreated or exposed to PD0325901. The percentage of apoptotic cells with subdiployd DNA is indicated for each condition and cell type. Standard deviations of the percentages are indicated for each condition. ** ≤ 0,01, *** ≤ 0,001 compared to untreated controls.

The encouraging activity of the MEK inhibitors used in the pathway inhibitor screening (see above) prompted us to study the antitumor effect of the MEK inhibitor PD0325901 on the melanospheres, based on its antitumor activity described in clinical studies [16]. Following 3 day-exposure to PD0325901, at doses comparable with those achieved *in vivo,* both wild type and mutated-BRAF cells displayed decreased proliferation/viability, with mutated-BRAF samples being more sensitive to the drug (Figure 3A). In order to distinguish the cytostatic from the cytotoxic effect and to unravel the molecular mechanisms of PD0325901 antitumor activity against malenospheres, we first performed cell cycle analysis of control and treated samples. After short exposure (2 days), PD0325901 greatly affected cell cycle progression by determining accumulation of cells in the G1 phase, both in the wild type and mutated-BRAF samples (Figure 3B). At the molecular level, together with a striking decrease in Cyclin D levels which is in line with the observed cell cycle arrest, treated samples displayed a decline in Erk and S6 phosphorylation, thus, proving MEK signaling inhibition by PD0325901 (Figure 3C). Given that PD0325901 may induce apoptosis in melanoma cell lines, we investigated whether a similar mechanism could account for the reduced number of viable cells in PD0325901-treated melanosphere samples [17]. Indeed, PD0325901-treated mutant-BRAF melanospheres contained a high fraction of apoptotic annexin V-positive cells compared to control samples. In contrast, PD0325901 treated wild type-BRAF melanospheres did not show such a dramatic increase (Figure 3D). Importantly, we found that both wild type and mutated-BRAF melanoma differentiated cells, were exquisitely sensitive to the drug, as indicated by the high fraction of sub-diploid cells detected in treated samples stained with Propidium Iodide (Figure 3E). This additional apoptosis assay confirmed that, at the level of melanospheres, only mutated-BRAF cells rapidly underwent PD0325901-induced apoptosis, while apoptotic hypodiploid DNA-cells were almost absent in the treated wild type-BRAF cells (Figure 3E). These results indicate that PD0325901 exerted strong cytotoxic activity against mutant-BRAF melanospheres, and a strong cytostatic activity against wild type-BRAF melanospheres, where cytotoxicity played a minor role. In contrast, differentiated melanoma cells were efficiently killed by PD0325901, regardless BRAF status (Figure 3E).

### Treatment with MEK inhibitor PD0325901 results in strong antitumor activity in melanosphere-derived xenografts

**Figure 4:**
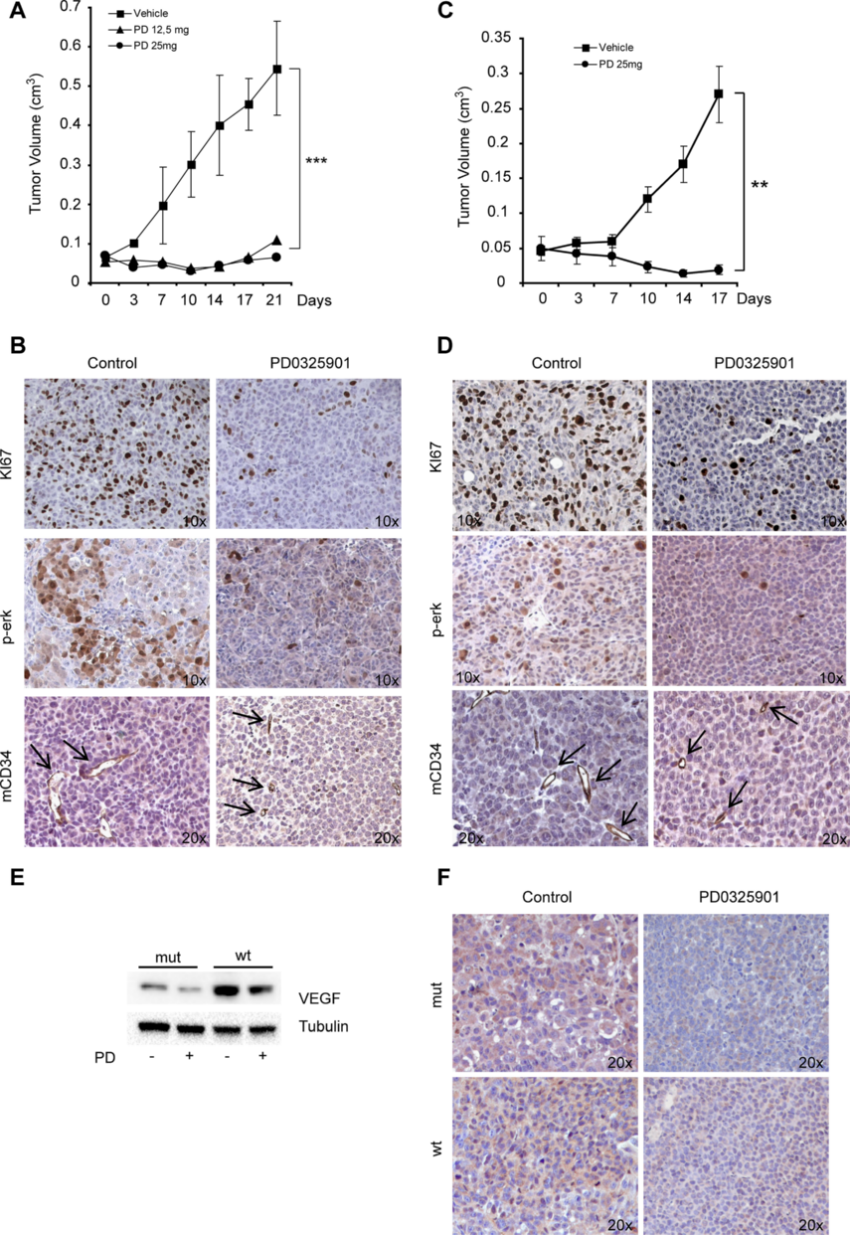
**Antitumor activity of PD in melanosphere-derived subcutaneous xenografts.** Growth curves of xenografts derived from mutant-BRAF **(A)** or wild type-BRAF **(C)** melanospheres in control or PD0325901-treated mice. Mean ± SD of 3 independent experiments is shown. *** p < 0,001. **B-D)** Immunohistochemistry for KI-67, p-Erk and mouse CD34 in control or treated BRAF-mutated **(B)** or BRAF-wild type **(D)** xenografts. **E)** Immunoblot for VEGF expression in control or PD0325901-treated representative melanospheres with mutated- or wild type-BRAF. **F)** Immunohistochemistry for VEGF in control or PD0325901-treated xenografts.

We investigated the activity of PD0325901 against melanosphere-generated subcutaneous xenografts. Doses of 25 or 12.5 mg/Kg were investigated in order to define a well tolerated dose with reduced toxicity and maximum antitumor activity, as the optimal doses and schedules for antitumor activity in the absence of toxicity was not previously determined in cancer patients. We chose the bi-weekly treatment schedule for drug administration based on previously published results showing high systemic toxicity occurring during daily drug administration [46] and as we previously experienced similar results in mice (results not shown). PD0325901 administration, by oral gavage, caused a striking reduction in tumor growth at both drug doses, displaying stronger activity for the higher dose (Figure 4A and Additional file 5: Figure S3A). Importantly, treated mice did not exhibit signs of toxicity under this treatment schedule. Immunoblot analysis of xenografts displayed markedly reduced levels of Erk and downstream S6 phosphorylation in treated tumors, indicating that PD0325901 levels reached *in vivo* were sufficient to achieve almost complete Erk inactivation and that the effects observed on tumors were caused by specific PD0325901 activity (Additional file 5: Figure S3B). Immunohistochemistry analysis of xenografts revealed decreased proliferation rates for treated tumors (lower Ki-67 expression in comparison with control tumors) and reduced activation of the Mek/Erk pathway (lower Erk phosphorylation) (Figure 4B). In addition, staining with murine CD34 antibody demonstrated a strong inhibitory effect of PD0325901 on tumor vascularization, as control tumors contained large vessels, while treated tumors displayed drastically compromised vasculature composed by minuscule vessels (Figure 4B). A decrease of tumor vascularization appeared also by macroscopic observation of the tumors (Additional file 5: Figure S3A). Importantly, similar results were obtained when xenografts were generated by wild type-BRAF melanospheres indicating that this strategy might constitute a potentially exploitable therapeutic approach both for mutated-BRAF and wild type-BRAF melanoma patients (Figure 4C and D).

Immunoblot analysis showed that VEGF levels were lower in treated-melanospheres (Figure 4E) and immunohistochemistry analysis showed that PD0325901-treated xenografts expressed reduced levels of VEGF in comparison with control tumors (Figure 4F). These results were obtained both for mutated BRAF and wild type BRAF melanospheres and xenografts and suggest that Mek inhibition might determine, together with a direct cytotoxic/cytostatic effect on tumor cells, a reduction of the tumor cell-dependent pro-angiogenic activity *in vivo.*

## Discussion

In the last years, several controversial findings concerning MIC has lead to intense investigation aiming at identifying and understanding the phenotype, frequency and behavior of these cells. Lately, a novel concept has emerged that partially modified the hierarchical organization model of tumors maintained by CSC, at least for some tumors, including melanoma. In contrast to the static and irreversible properties of CSC, this model proposes the existence of dynamic CSC that may arise from non stem tumor cells and possibly disappear upon microenvironmental stimuli [32,39]. Consequently, these CSC may display temporary changing phenotype and properties. This concept may partially explain the contradictory results that continue to emerge concerning MIC markers, frequency and tumorigenicity [40]. In fact, the identification of MIC based on marker expression has failed, so far, as suggested by the scarce agreement between different reports. Therefore, we used an alternative more reliable method for the isolation of tumorigenic melanoma cells relying on functional rather than phenotypic features based on the ability of undifferentiated tumor cells to grow as spheroid/aggregates, named tumor “spheres” in stem cell suitable culture conditions. This methodology provides cultures that are enriched in tumorigenic cells with CSC properties as we previously demonstrated for other tumors [41-44]. Highly tumorigenic cell-enriched populations were obtained without any prospective cell selection based on putative CSC-markers. This was done in order to circumvent the biased selection of cells relying on antigens endowed with weak CSC function or possibly undergoing dynamic temporal changes, as mentioned above. This system provided virtually unlimited amounts of highly tumorigenic cells from patient tumors that, besides carrying out a thorough investigation on their phenotype, nature, *in vitro* and *in vivo* properties necessary to accurately validate the experimental strategy, it allowed to investigate potential mechanisms of chemoresistance and potential strategies to overcome their aggressiveness through the inhibition of activated survival pathways. In agreement with other reports, we found little consensus with marker expression that was previously associated with putative MIC identified in different experimental conditions [38]. More importantly, all *in vitro* and *in vivo* functional assays supported the high stemness potential of melanospheres expanded *in vitro* (high proliferation, self renewal and multidifferentiative potentials, high tumorigenicity and ability to mimic the patient tumor in mice). They were highly chemoresistant even toward chemotherapeutic agents that were cytotoxic against differentiated cells and displayed a highly activated MAPK pathway, regardless of the BRAF mutational status. Thus, we used these highly valuable *in vitr*o and *in vivo* models to investigate the possibility to counteract melanoma aggressiveness by targeting the oncogenic MAPK pathway in these cells. Inhibition of Ras/RAF/MEK pathway, through the MEK inhibitor PD0325901, determined a stronger cytotoxic effect against mutant-BRAF melanospheres, while wild type-BRAF melanospheres mainly underwent growth inhibition upon MEK blockade. On the contrary, differentiated melanoma cells were exquisitely sensitive to MEK inhibition regardless BRAF status, undergoing massive apoptosis upon treatment. PD0325901 determined a strong antitumor efficacy in melanosphere-derived xenografts both with wild type or mutated BRAF. It is likely that the prompt and dramatic antitumor activity of MEK inhibition observed *in vivo*, both against mutated and wild type BRAF xenografts, might depend on the strong cytotoxicity of the drug against differentiated cells of both types. In addition, MEK inhibition determined a decreased VEGF production by melanospheres *in vitro* and a markedly reduced vascularization of tumors. This suggests that the antitumor effect of the drug *in vivo* may derive from both its direct toxicity on tumor cells and from a decreased production of the pro-angiogenic factor VEGF by tumor cells, hampering the production of tumor blood vessels. In line with these results, previous studies have shown that reduced VEGF expression was associated with inhibition of melanoma growth in mice [47].

Our results showed that PD0325901 antitumor activity was observed in both stem and non-stem cell populations, thus the proposed approach may represent a potentially successful therapeutic strategy against melanoma from both a classical hierarchical static model of CSC point of view and from a dynamic stemness perspective [48]. In fact, based on the recently proposed model of dynamic tumorigenic cells uncovering their ability to appear and disappear in different circumstances, it is clear that only a strategy that targets the stem and differentiated cells simultaneously may represent a potential tumor eradicating therapy. In fact, in this view, both stem and differentiated tumor cells need to be simultaneously depleted in order to avoid reappearance of the tumorigenic cells after interrupting stem cell-specific cytotoxic treatment [49,50].

Finally, a recent clinical trial reported evidence of PD0325901 systemic toxicity in treated patients [51]. Indeed, we observed toxicity in mice when followed a similar daily drug administration of high doses of MEK inhibitor (results not shown). In contrast, the twice a week low dose regimen did not cause toxicity in mice, while drastically affecting tumor growth, thus, indicating that optimization of the treatment schedule could lead to very promising results in patients. Notably, a recent phase III trial showed that treatment with a new MEK inhibitor (GSK1120212, GlaxoSmithKline) determined improved rates of progression-free and overall survival among patients who had metastatic melanoma with mutated BRAF, with very low toxicity [46]. In line with these clinical reports, we obtained significant activity when this drug was used against both tumorigenic and differentiated melanoma cells (Additional file 6: Figure S4). Importantly, we found that Mek inhibition *in vivo* determined a dramatic antitumor activity both in mutated- and wild type-BRAF tumors, suggesting that MEK inhibition, by different agents, might represent a powerful and safe strategy to counteract melanoma growth, thus improving patient outcome. However, considering the merely cytostatic activity exerted by MEK inhibitor against wild type BRAF melanoma stem-like cells *in vitro*, it may be possible that MEK inhibition might kill only the differentiated cells *in vivo,* as well, with consequent enrichemnt of tumors in stem-like cells. On the other hand, we found that tumors displayed reduced angiogenesis when treated with the drug, indicating an additional antitumor mechanism exerted by MEK inhibitor, besides the direct toxicity on tumor cells. Vasculature was dramatically compromised, with similar extent, in mutated and wild type BRAF xenografts, and most likely this event contributed to determine the dramatic inhibition of tumor growth observed in treated xenografts of both types. These results suggest that the marked antitumor activity of MEK inhibition may be mediated by multiple mechanisms *in vivo*, the direct cytotoxic or cytostatic activity against stem-like and differentiated tumor cells and the anti-angiogenic activity resulting from reduced tumor cell production of VEGF. The relative contribution of these two mechanisms might determine whether melanoma stem-like cells of wild type BRAF tumors are killed or spared by the treatment. Nevertheless, it may be possible that aggressiveness of both mutated and wild type tumors may increase following MEK inhibition, indicating an enrichment of treatment-resistant stem-like cells, similarly to what may occur during chemotherapy [52,53]. Even in this case, the possible enrichment of tumorigenic cells might be more limited in MEK-treated tumors in comparison with chemotherapy-treated tumors, as it might be counteracted by the anti angiogenic effect determined by Mek inhibition.

Finally, as MEK inhibition was highly cytotoxic for differentiated melanoma cells it is likely to hypothesize a combined treatment for wild type BRAF tumors with MEK inhibitors in association with differentiating agents. Hypothetically, this combination might lead to the exhaustion of stem-like cells that upon forced differentiation can be efficiently killed by the MEK inhibitor, with potential long term benefit for melanoma patients.

## Conclusions

The data presented in this study demonstrated that MEK inhibition determines a strong antitumor activity against the more tumorigenic metastatic melanoma cells expanded *in vitro* as melanospheres and against melanospheres-generated xenografts both with mutated or wild type BRAF. Although further studies are needed to clarify the long term effects of this approach, our findings suggest that, MEK inhibition, due to its multitargeting effect in vivo, might represent a therapeutic strategy with efficacy against the tumor-maintaining cells in metastatic melanoma, with potential relevance even in patients lacking BRAF mutation.

## Competing interests

The authors state no competing interests.

## Authors’ contributions

GS and AE conceived and designed the study. AE wrote the paper and GS contributed to the writing and to the critical reading of the paper. GS, KF, VS and FL performed the experiments. EP and ED provided patient samples and performed the immunohistochemistry. MB performed the flow cytometry analysis. LM, AP and DM contributed to the genetic characterization of melanospheres. MM contributed to critically revise the manuscript. RDM gave a key contribution to the intellectual content of the study. All authors read and approved the final manuscript.

## Supplementary Material

Additional file 1: Figure S1 *Phenotypic characterization of melanospheres.* A) Flow cytometric analysis of melanospheres for the indicated stem cell-associated antigens. White histograms are negative controls, grey histograms are specific antibody stainings. B) RT-PCR analysis for the expression of ABCB-5 in the following samples: (M) marker, melanospheres sample 1 to 5, melanocytes, positive control (lung cancer stem cells), negative control. C) RT-PCR analysis for the expression of Nanog and Oct-4 in the samples indicated as in B. D) Flow cytometric analysis of CD44 variant 6 in melanospheres, differentiated cells, fresh xenografts and melanocytes as indicated. Each type of cells was stained with unspecific antibody as negative control in the upper panels (control). Click here for file

Additional file 2: Figure S2 *In vitro stem cell properties of melanospheres.* A) *Proliferative potential of melanospheres*. Growth curve of melanospheres at early passages (kept in culture for few weeks after the isolation and before the experiment) or at late passages (after 6 month-culture). Cells were counted each week by trypan blue exclusion. B) *Self renewing ability (percentage of clonogenic cells) of melanospheres.* Percentage of cells able to form new spheres after single cell plating in limiting dilution analysis for the indicated samples (first panel). Percentage of self-renewing cells obtained from primary, secondary or tertiary spheres in limiting dilution analysis (second panel). Percentage of self renewing in undifferentiated (spheres) or differentiated cells obtained under stem cell culture conditions (undifferentiative) or under differentiative conditions as indicated (third panel). Comparison of self-renewing cells in cells previously expanded under stem cell conditions (SC medium) or under standard conditions for differentiated melanoma cells (RPMI) (last panel). The values represent mean +/- SD of three independent experiments. Student’ s T test was used to determine p-value (*p<0,1; **p<0,01; ***p<0,001). C) *Multidifferentiation potential of melanospheres.* (left) Melanogenic differentiation (S-100); (middle) Adipogenic differentiation (Oil-red-O); (right) Osteogenic differentiation (Alcaline Phosphatase activity). Click here for file

Additional file 3: Table S1 Clinical Staging of melanomas and analysis of genetic status of the NRAS, BRAF, PTEN and GNAQ genes in melanospheres. Click here for file

Additional file 4 Analysis of genetic status of the NRAS, BRAF, PTEN and GNAQ genes in melanospheres. Click here for file

Additional file 5: Figure S3 *Antitumor activity of PD in melanosphere-derived subcutaneous xenografts.* Tumor images (A) and immunoblot for pathway activation (B) of melanosphere-derived xenografts obtained from control or PD0325901-treated mice. Click here for file

Additional file 6: Figure S4 *Mek inhibition by GSK1120212*. A) Three thousand cells obtained from melanosphere dissociation were plated in 96-well flat-bottom plates and Mek inhibitor GSK1120212 (Glaxo Smith Kline) was added at the indicated doses. Cell viability was evaluated after 3 days treatment by luminescent cell viability assay (CellTiter-Glo, Promega, Madison, WI, USA). B) Stem versus differentiated melanoma cells (as indicated) were treated as in A for comparison of Mek inhibitor activity against the different cell types. Data represented are mean of three independent experiments performed with the two experimental procedures. Student’ s T test was used to determine p-value (**p<0,01; ***p<0,001). Click here for file
