# Supplementary figures and images for: Mek inhibition results in marked antitumor activity against metastatic melanoma patient-derived melanospheres and in melanosphere-generated xenografts
Source: J Exp Clin Cancer Res. 2013 Nov 16;32(1):91. doi: 10.1186/1756-9966-32-91 (PMC3874650; doi:10.1186/1756-9966-32-91)

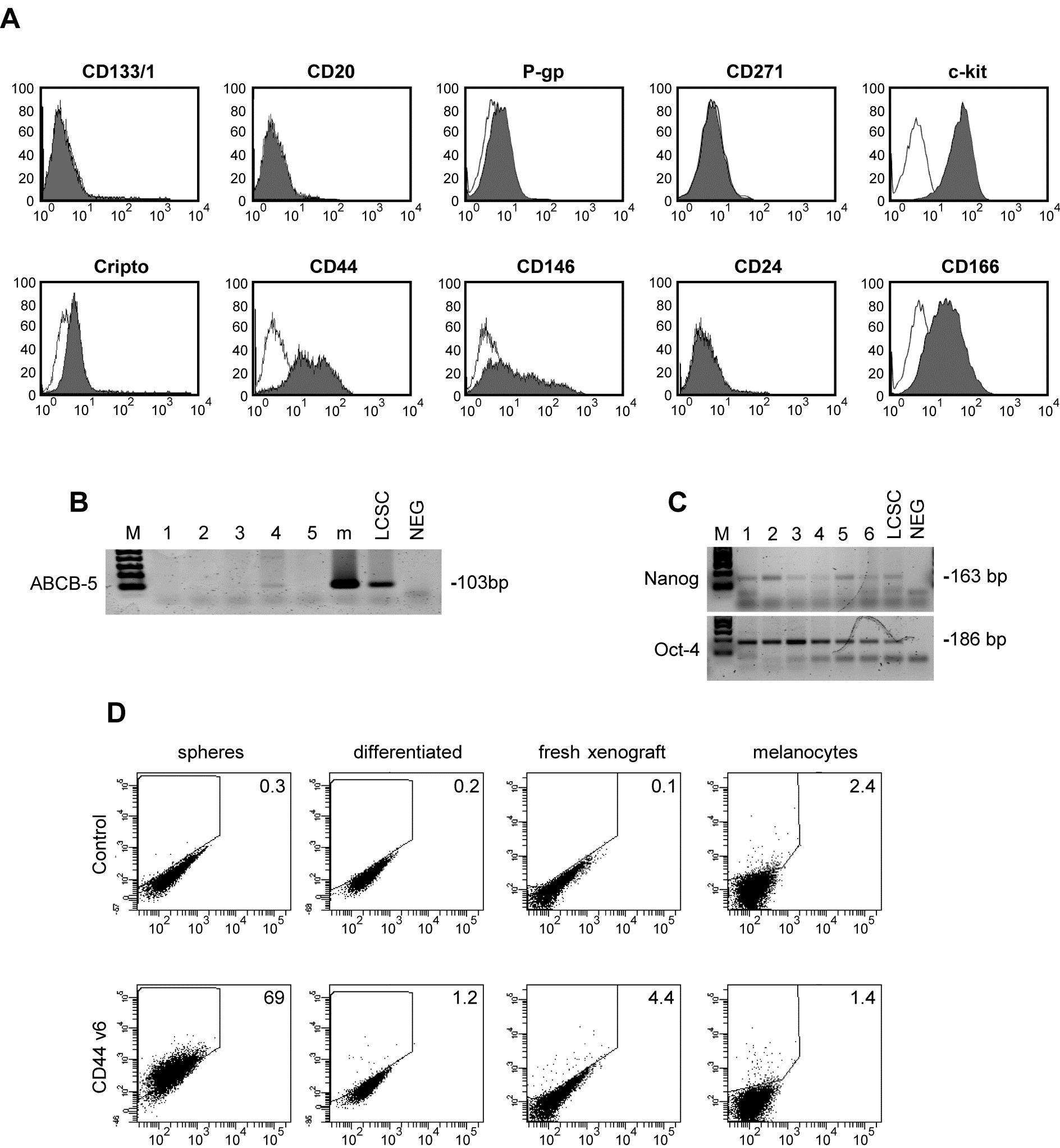

Supplement: Additional file 1: Figure S1 — Phenotypic characterization of melanospheres. A) Flow cytometric analysis of melanospheres for the indicated stem cell-associated antigens. White histograms are negative controls, grey histograms are specific antibody stainings. B) RT-PCR analysis for the expression of ABCB-5 in the following samples: (M) marker, melanospheres sample 1 to 5, melanocytes, positive control (lung cancer stem cells), negative control. C) RT-PCR analysis for the expression of Nanog and Oct-4 in the samples indicated as in B. D) Flow cytometric analysis of CD44 variant 6 in melanospheres, differentiated cells, fresh xenografts and melanocytes as indicated. Each type of cells was stained with unspecific antibody as negative control in the upper panels (control). [file 1756-9966-32-91-S1.tiff]

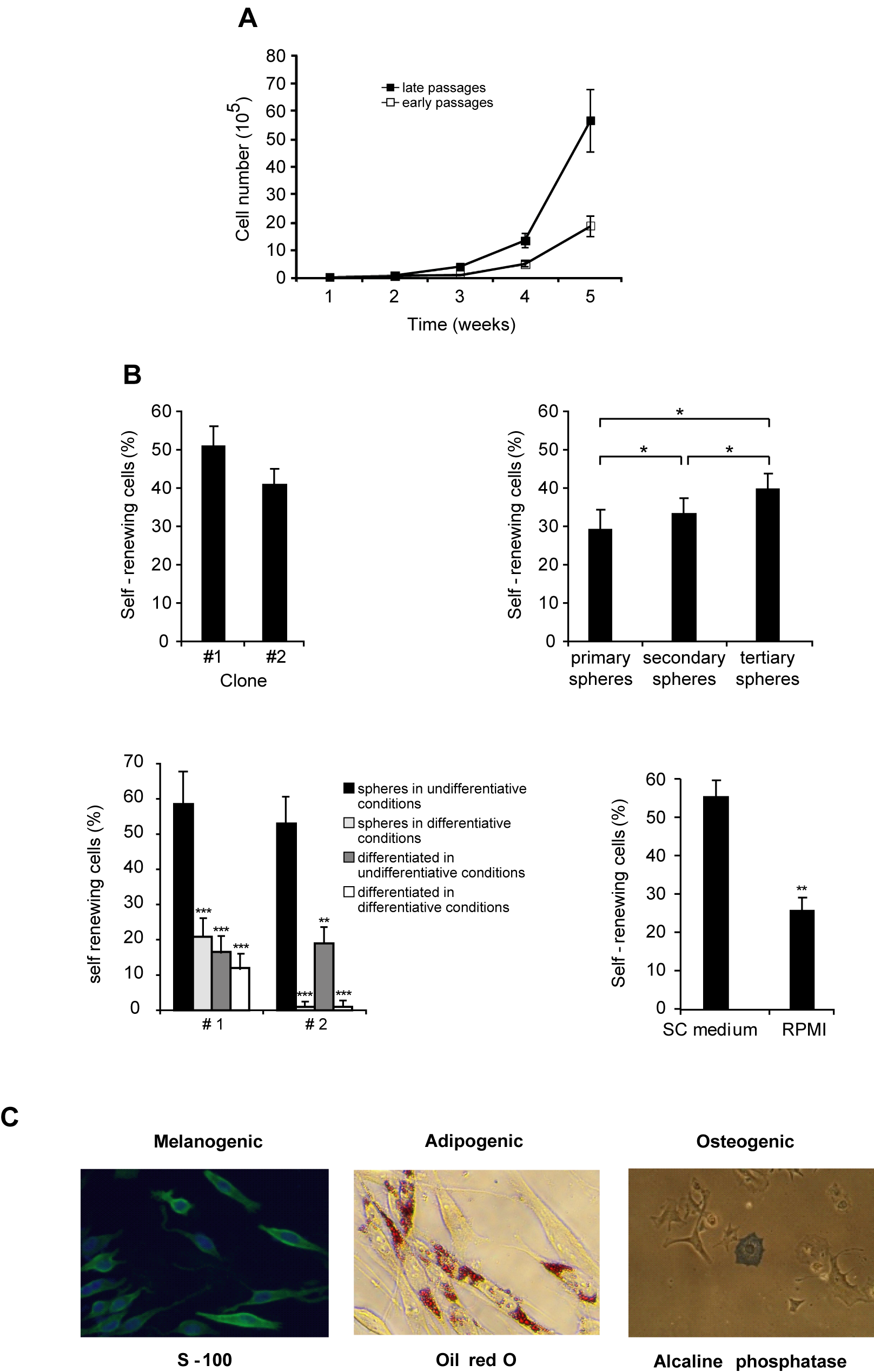

Supplement: Additional file 2: Figure S2 — In vitro stem cell properties of melanospheres. A) Proliferative potential of melanospheres. Growth curve of melanospheres at early passages (kept in culture for few weeks after the isolation and before the experiment) or at late passages (after 6 month-culture). Cells were counted each week by trypan blue exclusion. B) Self renewing ability (percentage of clonogenic cells) of melanospheres. Percentage of cells able to form new spheres after single cell plating in limiting dilution analysis for the indicated samples (first panel). Percentage of self-renewing cells obtained from primary, secondary or tertiary spheres in limiting dilution analysis (second panel). Percentage of self renewing in undifferentiated (spheres) or differentiated cells obtained under stem cell culture conditions (undifferentiative) or under differentiative conditions as indicated (third panel). Comparison of self-renewing cells in cells previously expanded under stem cell conditions (SC medium) or under standard conditions for differentiated melanoma cells (RPMI) (last panel). The values represent mean +/- SD of three independent experiments. Student’ s T test was used to determine p-value (*p<0,1; **p<0,01; ***p<0,001). C) Multidifferentiation potential of melanospheres. (left) Melanogenic differentiation (S-100); (middle) Adipogenic differentiation (Oil-red-O); (right) Osteogenic differentiation (Alcaline Phosphatase activity). [file 1756-9966-32-91-S2.tiff]

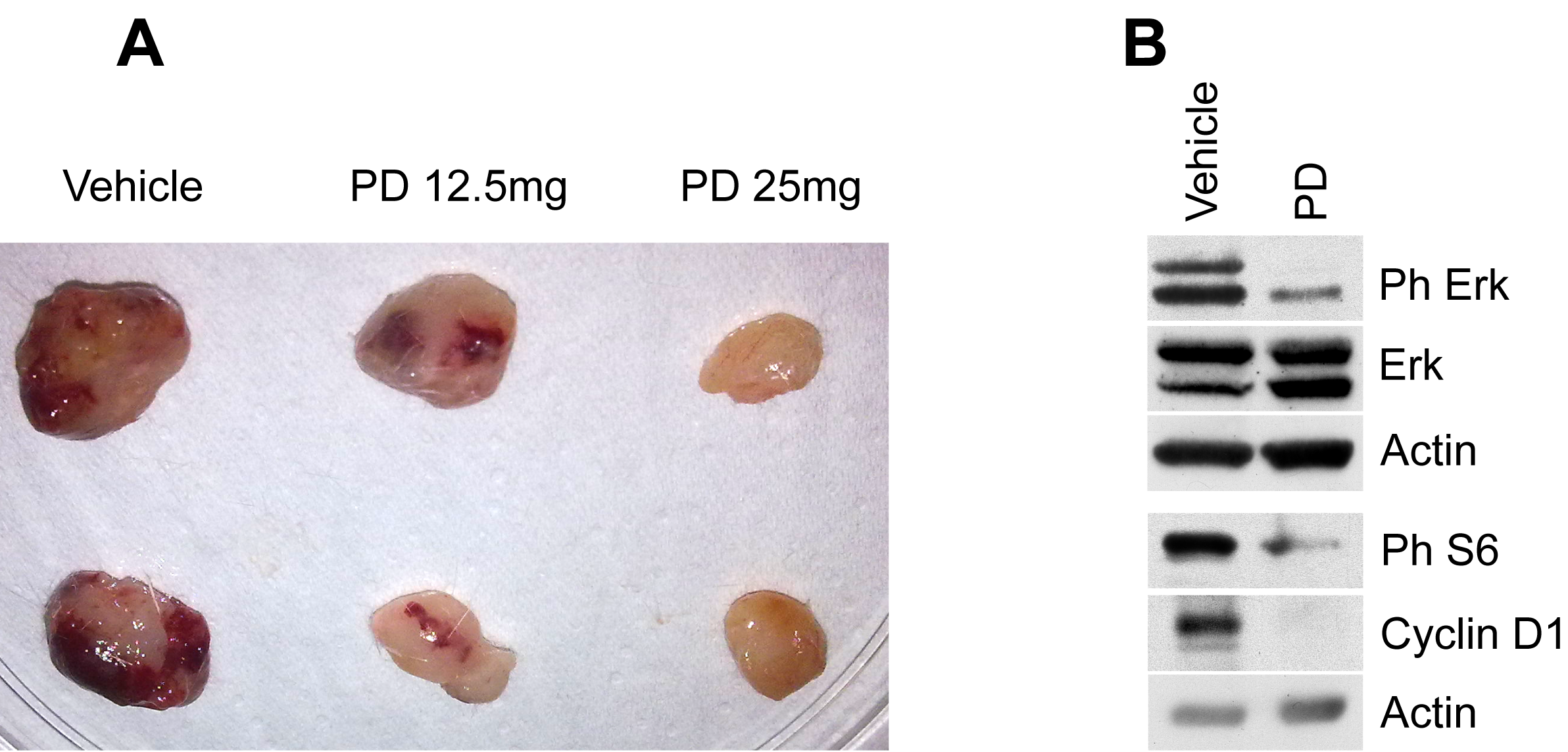

Supplement: Additional file 5: Figure S3 — Antitumor activity of PD in melanosphere-derived subcutaneous xenografts. Tumor images (A) and immunoblot for pathway activation (B) of melanosphere-derived xenografts obtained from control or PD0325901-treated mice. [file 1756-9966-32-91-S5.tiff]

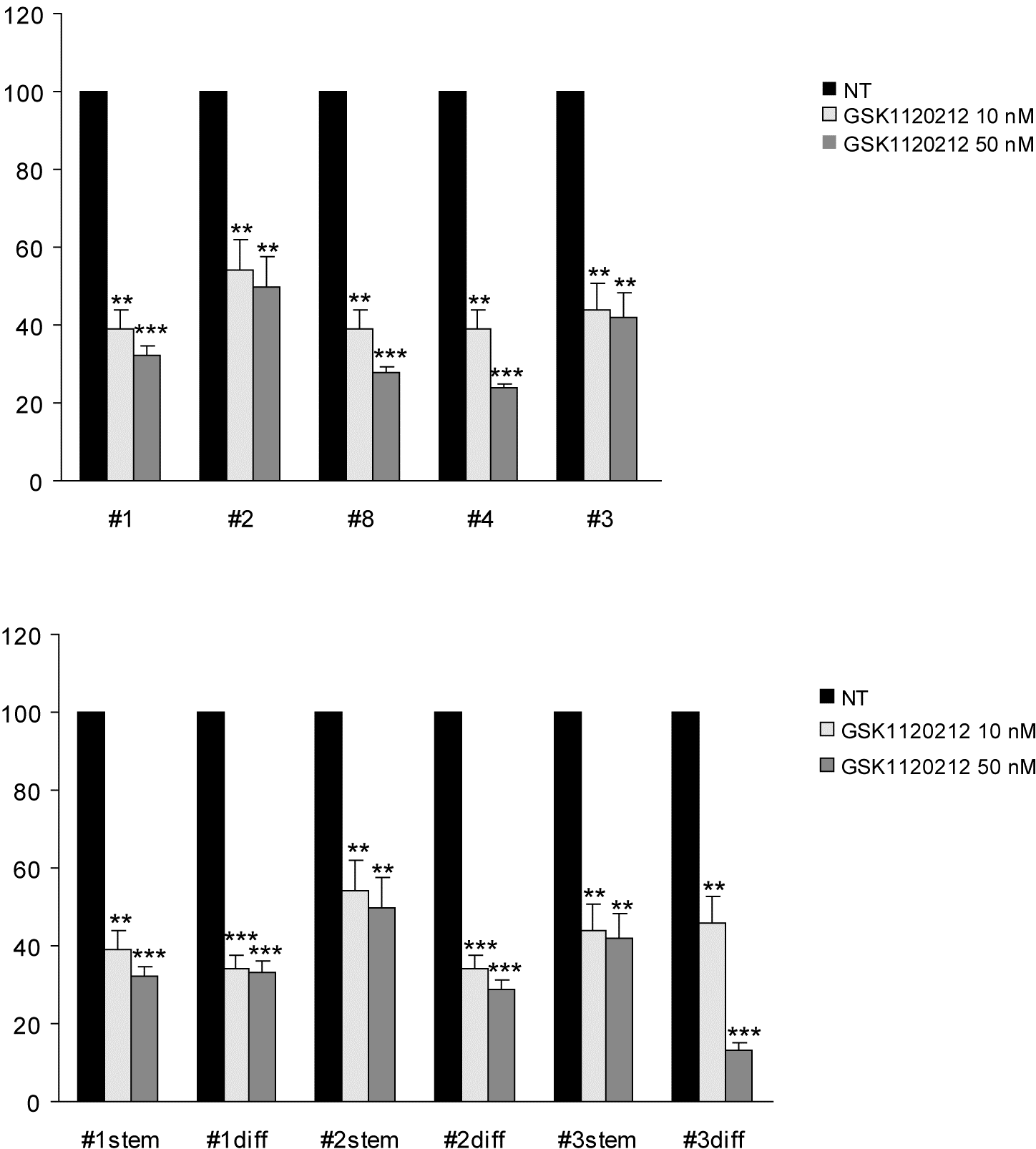

Supplement: Additional file 6: Figure S4 — Mek inhibition by GSK1120212. A) Three thousand cells obtained from melanosphere dissociation were plated in 96-well flat-bottom plates and Mek inhibitor GSK1120212 (Glaxo Smith Kline) was added at the indicated doses. Cell viability was evaluated after 3 days treatment by luminescent cell viability assay (CellTiter-Glo, Promega, Madison, WI, USA). B) Stem versus differentiated melanoma cells (as indicated) were treated as in A for comparison of Mek inhibitor activity against the different cell types. Data represented are mean of three independent experiments performed with the two experimental procedures. Student’ s T test was used to determine p-value (**p<0,01; ***p<0,001). [file 1756-9966-32-91-S6.tiff]
