# Supplementary material for: Mek inhibition results in marked antitumor activity against metastatic melanoma patient-derived melanospheres and in melanosphere-generated xenografts
Source: J Exp Clin Cancer Res. 2013 Nov 16;32(1):91. doi: 10.1186/1756-9966-32-91 (PMC3874650; doi:10.1186/1756-9966-32-91)
Supplement: Additional file 3: Table S1 — Clinical Staging of melanomas and analysis of genetic status of the NRAS, BRAF, PTEN and GNAQ genes in melanospheres. [file 1756-9966-32-91-S3.pdf]

	TNMstage	NRAS	BRAF	PTEN	GNAQ (exon 4-5)
melanoma 1	lung met	wt	V600E	wt	wt
melanoma 2	lung met	wt	V600E	wt	wt
melanoma 3	lung met	wt	WT	wt	wt
melanoma 4	Lymph node met	wt	V600E	wt	wt
melanoma 5	Subcutaneous met	wt	V600K	wt	wt
melanoma 6	Subcutaneous met	wt	WT	wt	wt
melanoma 7	Subcutaneous met	wt	WT	wt	wt
melanoma 8	Subcutaneous met	wt	V600K	wt	wt
