# Supplementary material for: Mek inhibition results in marked antitumor activity against metastatic melanoma patient-derived melanospheres and in melanosphere-generated xenografts
Source: J Exp Clin Cancer Res. 2013 Nov 16;32(1):91. doi: 10.1186/1756-9966-32-91 (PMC3874650; doi:10.1186/1756-9966-32-91)
Supplement: Additional file 4 — Analysis of genetic status of the NRAS, BRAF, PTEN and GNAQ genes in melanospheres. [file 1756-9966-32-91-S4.doc]

**Additional file 4**

**Analysis of genetic status of the NRAS, BRAF, PTEN and GNAQ genes in melanospheres.** DNA was extracted with Pure Link Genomic DNA purification kit purchase from Invitrogen (Gibco-Invitrogen, Carlsbad, CA). For detection of BRAF mutations the following primers were used: BRAF Forward-TCA TAA TGC TTG CTC TGA TAG GA and Reverse-GGC CAA AAA TTT AAT CAG TGG A. This primers produced an amplicon of approximately 240bp. To detect the NRASQ61R mutation Exon 2 of NRAS was PCR amplified with the following forward GTT ATA GAT GGT GAA ACC TG and reverse ATA CAC AGA GGA AGC CTT CG primers. The amplicon was 115bp. For all PCR reactions, 100 ng of extracted DNA were used with the following PCR conditions: 96°C for 10 minutes, followed by 45 cycles of 96°C denaturation for 40 seconds, annealing at 51°C for 90 seconds, extension at 72°C for 90 seconds, and a final extension of 10 minutes at 72°C. PCR products were loaded on 2% agarose gel for quality control and 30ng of each PCR sample used for sequence analysis MWG (Ebersberg,Germany). Sequencing of PTEN exons 1 to 9, was done by PCR amplification and direct sequencing of both strands for all samples as previously described (Sa*al et a*l., 2005) using a 3500 Genetic Analyzer (Applied Biosystems, Foster City, CA). Mutation analysis of GNAQ exon 4 and 5 was carried out by direct sequencing of amplified PCR products of exons 4 ad 5. The following primers were used: exon 4 forward 5’TTAATGACTTGGACCGCGT, exon 4 reverse 5’ACAGGGATCATCGAATACCCC; exon 5 forward 5’ ACAGGGATCATCGAATACCCC and exon 5 reverse 5’AGTTCTCGTGGAGTCAGACA. Primers were purchased from Roche Dagnostics Spa, Monza, Italia. PCR was performed using 50 ng genomic DNA as template. Each mixture contained 8 pmol of each primer. The reactions were performed in 1× GeneAmp 10X PCR Buffer II (Applied Biosystems), 0.25 μmol/L dNTPs, 2 mmol/L MgCl2 solution, and 1.25 U AmpliTaq DNA Polymerase (Applied Biosystems, CA USA).

The amplification reactions were as follows: an initial denaturation cycle of 95˚C for 5 min; 45 cycles of denaturation (95˚C for 30 s), annealing (64˚C for 30 s for exon 4 and 55˚C for 30 s for exon 5), and elongation (72˚C for 1 min) ; and a final extension cycle at 72˚C for 5 min. The PCR products were purified with 1 ml ExoI/SAP (37˚C for15 minutes, then 85˚C for 15 minutes) and were then sequenced directly on both strands using the BigDyeH Terminator v1.1 cycle sequencing kit (Applied Biosystems) according to manufacturer’s protocol and analysed by the ABI 3500 Genetic Analyzer (Applied Biosystem, CA, USA).

**In vitro differentiation of melanospheres in mesenchymal lineages.** Osteogenic and adipogenic differentiation were obtained using the hMSC differentiation bullet Kit-Osteogenic or –Adipogenic in accordance with the manufacturer’s instructions (Lonza, East Rutherford, NJ, USA). The acquisition of differentiation markers was evaluated by visible chemical/stain reaction with Oil red O (Sigma-Aldrich, St. Louis, Mo, USA) for adipogenic, with Alkaline Phosphatase substrate kit III (Vector Laboratories, Burlingame, CA, USA) for osteogenic or by immunofluorescence for S100 for melanocyte differentiation.

**Flow cytometry and immunofluorescence.** For flow cytometry, tumor-spheres were dissociated as single cells, washed and incubated with the appropriate dilution of control or specific antibody. Antibodies used were: PE-conjugated anti-CD133/1, from Miltenyi (Bergisch Gladbach, Germany), FITC-conjugated anti-CD44, anti-CD24 anti-CD20, anti-CD166, and PE-conjugated anti-CD146, anti-CD271 and PE/Cy5-conjugated anti c-Kit (BD, Pharmingen, San Josè, CA), PE-conjugated anti-CD44 Variant 6 and anti-CRIPTO (R&D system, Minneapolis, MN), anti-Pgp clone mm.4.17 Chemicon (Billerica, MA USA), anti-S100 (DAKO, Glostrup, Denmark). After 45 minutes incubation cells were washed, where necessary incubated with FITC-conjugated rabbit anti-mouse secondary antibodies (Molecular Probes, Eugene, OR) for 30 minutes, washed again, suspended in PBS containing 7AAD (10 μg/ml) and analyzed with a FACScan (Becton Dickinson. For immunofluorescence studies cells were grown on poly-D-lysine-coated glass coverslips, fixed with 2% paraformaldehyde for 20 min at 37°C and permeabilized with 0.1% Triton X-100/ PBS for 3 min at room temperature before incubation with the specific or control antibody. Stained cells were visualized with an Olympus confocal microscope.

**Cell proliferation and Self renewal assay.** Spheres were plated at 10,000 cells/ml in growth medium supplemented with growth factors and after extended mechanical dissociation of culture aliquots, single cells were counted by trypan blue exclusion once a week. To determine their self-renewal ability, sphere-forming or differentiated melanoma cells were seeded in 96-well plates at single cell per well density. Shortly after seeding, single cell-containing wells were identified and analyzed for the ability to generate long-term growing spheres whose expansion was stable for more than 5 months. Soft agar colony-forming assay was carried out as previously described (Bartuc*ci et a*l., 2012). Briefly 500 single cells were plated in the top agar layer in each well of a 24-well culture plate with 0.25% top agar layer and 0.5% bottom agar layer (SeaPlaque Agarose, Cambrex, NJ, USA). Cells were cultivated at 37 °C for 3 weeks in stem cell- or differentiative culture conditions. Colonies from triplicate wells were stained with crystal violet (0.01% in 10% MetOH), visualized and counted under microscope.

**Reverse Transcription-PCR Analysis.** Total RNA was extracted using RNeasy Mini kit Qiagen (Hilden, Germany) according to the manufacturer’s instructions. RNA concentration was determined and quality was assessed by 1% agarose gel electrophoresis. RNA (1 μg) was reverse transcribed into cDNA by using SuperScript II RT with oligo(dT) as primer (Invitrogen, Grand Island, NY) according to the manufacturer’s protocol. The following primers were used for RT-PCR: ABCB5 Forward ATG TAC AGT GGC TCC GTT CC and Reverse ACA CGG CTG TTG TCA CCA TA (103bp amplicon); Nanog Forward GCT GAG ATG CCT CAC ACG GAG and Reverse TCT GTT TCT TGA CTG GGA CCT TGT C. (163bp amplicon); Oct 3/4 Forward TGG AGA AGG AGA AGC TGG AGC AAA A and Reverse GGC AGA TGG TCG TTT GGC TGA ATA (186bp amplicon). For PCR reactions, 2 μl of cDNA were amplified under the following conditions: 30 cycles for ABCB5 (Ta 60°C), 35 cycles for Nanog and Oct 3/4 (Ta 55°C). PCR products were analyzed on 2% agarose gel.

**References**

1. Saal LH, Holm K, Maurer M, Memeo L, Su T, et al. (2005) PIK3CA mutations correlate with hormone receptors, node metastasis, and ERBB2, and are mutually exclusive with PTEN loss in human breast carcinoma. Cancer Res 65: 2554-2559.

2. Bartucci M, Svensson S, Romania P, Dattilo R, Patrizii M, et al. (2012) Therapeutic targeting of Chk1 in NSCLC stem cells during chemotherapy. Cell Death Differ 19: 768-778.
